# Building an intelligent brain platform for small and medium-sized enterprises using ChatGLM and Multi-Agent Systems

**DOI:** 10.1371/journal.pone.0340964

**Published:** 2026-03-27

**Authors:** Daohong Yuan

**Affiliations:** NongFu Store Development Group Co., Ltd, Beijing, China; West Pomeranian University of Technology, POLAND

## Abstract

Large language models (LLMs) have demonstrated strong capabilities in semantic understanding and text generation. However, their direct application in the segmented and specialized domains of small and medium-sized enterprises (SMEs) presents several challenges. These include semantic overgeneralization, poor alignment with enterprise-specific knowledge, and insufficient domain expertise. To address these limitations, this study proposes an “Enterprise Intelligent Brain” platform tailored to the business needs of SMEs. The platform is built upon Chat Global Language Model (ChatGLM) and is enhanced through a multi-agent coordination mechanism and structured support from enterprise knowledge graphs. The study centers on improving the platform’s semantic adaptability and intelligent responsiveness in real-world enterprise scenarios. It begins by identifying the core semantic demands of typical SME operations—such as policy consultation, customer service, and business process execution—and constructs a triadic system architecture that integrates three key components: semantic parsing, task scheduling, and knowledge support. Methodologically, the platform applies domain-specific fine-tuning to the ChatGLM model to enhance relevance and precision. It also incorporates a multi-agent task allocation framework and utilizes knowledge graph reasoning to improve contextual accuracy and domain knowledge integration. The effectiveness of the proposed system is evaluated using three public datasets: Baidu DuReader-Enterprise, the E-commerce Dialogue Dataset, and the Enterprise Knowledge Graph-Based Q&A Dataset. Experimental results confirmed that the optimized system significantly outperformed the baseline model across multiple metrics. Notably, it achieved a task completion rate of up to 99.904%, an average response time as low as 0.858 seconds, a context retention score of up to 0.953, and a user satisfaction rating of up to 4.767. Additionally, the system demonstrated strong performance in knowledge invocation coverage and error recovery, indicating its robustness in complex and dynamic SME environments. Therefore, this study provides a practical and scalable framework for deploying LLMs in domain-specific SME contexts. It offers both a technical solution and theoretical insights for developing enterprise-grade semantic intelligence platforms capable of supporting intelligent decision-making and service automation.

## Introduction

The global digital economy is advancing rapidly, driven by innovations in artificial intelligence (AI), big data, and cloud computing. These technologies are accelerating the intelligent transformation of enterprises. Large enterprises, with their extensive resources and technical expertise, have made significant progress in building integrated systems for decision-making, customer service, and operational optimization [1,2]. In contrast, small and medium-sized enterprises (SMEs) often face constraints related to technology infrastructure, financial investment, and specialized personnel. As a result, their digital transformation and adoption of intelligent services have lagged behind. To address this gap, SMEs urgently require cost-effective, adaptable, and targeted “lightweight intelligent platforms” to enhance their management capabilities and market competitiveness [3]. The emergence of pre-trained language models has opened new avenues for developing high-quality enterprise intelligence platforms. Among these, Chat Global Language Model (ChatGLM), the leading Chinese large language models (LLMs), demonstrates strong capabilities in Chinese language understanding, contextual reasoning, and multi-turn dialogue generation. These features enable it to support a variety of enterprise-oriented intelligent services, including intelligent question answering, text generation, and semantic analysis [4]. Furthermore, its open-source nature, support for fine-tuning, and compatibility with local deployment make it well-suited for building secure and customizable intelligent systems for SMEs [5]. In parallel, multi-agent systems (MAS) have been widely adopted in domains such as manufacturing scheduling, financial decision-making, and information retrieval. MAS architectures enable autonomous collaboration, task delegation, and distributed processing, making them ideal for managing complex enterprise environments. When integrated into intelligent service platforms, MAS can significantly enhance capabilities in multi-task processing, scenario adaptability, and self-learning. This results in a “business division + intelligent collaboration” service model that provides systemic, intelligent support [6,7]. Given this context, integrating ChatGLM with MAS to construct an “Enterprise Intelligent Brain” platform tailored for SMEs holds both theoretical and practical significance. This integrated platform aims to overcome the limitations of traditional systems in terms of interactivity and intelligence. By enabling natural language-driven business processes, intelligent knowledge question and answer (Q&A), and context-aware recommendations, the platform supports a more responsive and scalable enterprise environment. The MAS further enhances its robustness and flexibility, allowing the platform to adapt to a wide range of dynamic business scenarios and meet the evolving needs of SMEs [8].

Accordingly, this study proposes a novel intelligent platform for SMEs that combines the semantic capabilities of ChatGLM with the collaborative architecture of MAS. Through systematic technological integration, architectural design, and experimental validation, the platform is evaluated for its effectiveness in intelligent decision support, knowledge services, and collaborative task management. The ultimate goal is to empower SMEs with efficient, scalable, and sustainable intelligent transformation.

## Literature Review

With the rapid advancement of AI, technologies such as LLMs and MAS are being increasingly adopted in enterprise management, intelligent decision-making, and customer service. Researchers around the world have explored the construction of intelligent enterprise platforms from various perspectives. Their work primarily focuses on three areas: analyzing enterprise intelligence requirements, investigating the practical applications of LLMs, and enhancing the functional capabilities of MAS. Alhari and Fajrillah (2022) pointed out that SMEs had a high demand for intelligent platforms that were frequently usable, cost-efficient, and highly adaptable. However, most mainstream solutions were developed for large enterprises and lacked a general-purpose service model suitable for SMEs [9]. Yathiraju (2022) highlighted that SMEs often faced two major obstacles: cognitive limitations and the absence of appropriate tools for management, customer support, and knowledge acquisition. While intelligent platforms had the potential to lower cognitive costs, their implementation and deployment remained overly complex [10]. Khan et al. (2024) provided empirical evidence that Chinese LLM-powered Q&A systems significantly improved accuracy and response speed in public service scenarios. Their findings underscored the strong capabilities of pre-trained models in Chinese language understanding and question generation [11]. Wang et al. (2024) further emphasized that ChatGLM, due to its lightweight deployment and adaptability to specific domains, performed well in enterprise tasks such as knowledge-based Q&A, text summarization, and semantic classification [12]. Yang et al. (2025) proposed that the integration of LLMs with knowledge graphs significantly enhanced the accuracy and efficiency of enterprise intelligent Q&A systems. By combining the natural language processing capabilities of LLMs with the structured knowledge representation of knowledge graphs, enterprises can achieve more precise knowledge reasoning and contextual understanding, thereby improving customer service quality and response speed [13]. Arora (2025) reported that the combined application of LLMs and knowledge graphs also strengthened enterprise decision-support systems. By providing LLMs with real-time, domain-specific knowledge, knowledge graphs enabled efficient knowledge flow and management during complex decision-making processes, effectively improving the system’s overall intelligence level [14].

Existing research offers valuable theoretical insights and technical references for the development of intelligent enterprise platforms. However, significant gaps remain—particularly in the context of platforms tailored to SMEs. Current solutions often lack the lightweight design and universality required for SME adoption. Moreover, while LLMs have been explored in enterprise applications, most studies focus solely on semantic processing, with limited attention to their integration into system-level, multi-task scenarios. To address these limitations, this study integrates the ChatGLM large language model with a MAS, forming a hybrid architecture that combines semantic understanding with collaborative processing. This “intelligent brain” platform is specifically designed to meet the low-resource, high-flexibility demands of SMEs. It supports local deployment, fine-tuning, and rapid adaptation, making it a practical and scalable solution for dynamic enterprise environments.

## Research design

### Platform demand analysis and functional positioning

SMEs typically face diverse and rapidly changing management and service needs, especially amid ongoing digital transformation [15,16]. Compared to large enterprises, they often struggle with limited technological infrastructure, constrained budgets, and a shortage of specialized personnel. These challenges hinder the implementation of complex intelligent systems. Therefore, the development of a cost-effective, easy-to-deploy intelligent platform—capable of handling intelligent Q&A and collaborative task processing—is of considerable practical significance. Based on the literature review, the common operational needs of SMEs can be categorized into several core areas, as summarized in Table 1:

**Table 1:** Platform demand analysis.

Demand	Analysis
Information Retrieval and Knowledge Q&A	Employees in SMEs often require access to information across areas such as finance, HR, legal compliance, and product details. Traditional document-based searches are inefficient and offer limited coverage. There is an urgent need for intelligent tools with semantic understanding and Q&A capabilities [17].
Task Scheduling and Business Collaboration	In scenarios such as customer service, order processing, and after-sales support, SMEs require systems capable of logical task judgment and distribution to either replace or assist manual operations [18].
Internal Knowledge Management	Knowledge within SMEs is often fragmented and lacks structured organization or centralized management, leading to information silos and potential knowledge loss. Constructing a knowledge graph is crucial for knowledge integration, organization, and intelligent access.
Lightweight Deployment and Low-Barrier Operations	With limited IT infrastructure, SMEs need platforms that are resource-efficient, easy to deploy, and user-friendly. Support for local deployment or one-click cloud integration is essential to reduce operational thresholds [19,20].

Based on the preceding analysis, this study proposes a smart enterprise platform designed for SMEs, integrating the ChatGLM language model with a MAS. The platform’s functional architecture is organized as follows:

1. Intelligent Q&A and Semantic Parsing Module: Leverages the ChatGLM model to support natural language understanding, regulatory interpretation, enterprise document queries, and guided business operations.
2. Multi-Agent Collaborative Scheduling Module: Utilizes a multi-agent framework to identify, allocate, and execute internal enterprise tasks, enabling closed-loop task management and coordination.
3. Knowledge Graph-Driven Module: Builds customized enterprise knowledge graphs from business data to enhance semantic parsing, logical inference, and knowledge abstraction.
4. Feedback Learning and Adaptive Optimization Module: Collects user interaction data to refine model parameters and agent strategies, continually improving the platform’s ability to deliver personalized services [21,22].
5. Lightweight Deployment Module: Supports flexible deployment options including on-premises and private cloud solutions. It enables API integration, SaaS delivery, and ensures system controllability, security, and ease of integration.

In summary, the platform integrates four essential capabilities: semantic understanding, task collaboration, knowledge management, and user accessibility. It is designed to be low-threshold, highly adaptable, and capable of continuous evolution, providing intelligent and efficient support for enterprise operations and decision-making.

### Overall platform architecture design

To address the broad needs of SMEs—including intelligent Q&A, business collaboration, knowledge management, and flexible deployment—this study presents a unified platform architecture, referred to as the “Enterprise Intelligent Brain.” The architecture combines ChatGLM with a MAS in a modular design. It is driven by natural language understanding, powered by intelligent agents, and supported by a structured knowledge graph. The system enables intelligent coordination and dynamic service responses across various tasks, roles, and business scenarios. The platform is structured into five core layers: Data Access Layer, Semantic Understanding Layer, Multi-Agent Collaboration Layer, Knowledge Service Layer, and Platform Support Layer. A detailed breakdown of this architecture is provided in Table 2.

**Table 2:** Logical Analysis of Platform Architecture.

Framework	Analysis
Data Access Layer	Responsible for integrating raw data from internal enterprise systems, business interface endpoints, and external open datasets.
Semantic Understanding Layer	Serves as the core for natural language interaction, leveraging the ChatGLM large language model for in-depth semantic parsing and intent recognition.
Multi-Agent Collaboration Layer	Establishes a network of diverse agents with specialized roles, including information agents, scheduling agents, knowledge retrieval agents, and feedback optimization agents.
Knowledge Service Layer	Structures enterprise knowledge into a graph-based format using an entity–relation–attribute triplet database.
Platform Support Layer	Provides unified system-level support functions to ensure operational stability and scalability.

The platform adopts a tri-layered architecture composed of semantic understanding, multi-agent collaboration, and knowledge graph reasoning. This integrated design forms a smart enterprise service system specifically tailored for SMEs. The architecture not only enables rapid and natural user interaction but also delivers strong adaptability to diverse tasks, flexible service orchestration, and ongoing system evolution. As a result, it functions as a reliable, efficient, and intelligent decision-support tool for SME operations.

### Core module design and key mechanisms

To enhance the platform’s intelligence and scalability in semantic processing, task coordination, and knowledge reasoning, the study applies a model-driven design approach to its three core modules. Mathematical formulations are used to describe the internal mechanisms and operational logic of the following components: the Semantic Parsing Module, the Intelligent Agent Task Allocation Mechanism, and the Knowledge Graph Reasoning Module. These formulations establish the theoretical foundation for model integration and task optimization across the system.

The Semantic Parsing Module, built upon the ChatGLM model, is responsible for encoding input sentences into semantic vectors and decoding them into meaningful outputs. It leverages embedding and attention mechanisms to extract and preserve contextual semantics for downstream tasks.


$$H=\text{Transformer}(E(X))=\{h_{1},h_{2},\ldots ,h_{T}\}$$ (1)

In Equation (1), H denotes the semantic vector, E(X) represents the word embedding vector, and $h_{1},h_{2},\ldots ,h_{T}$ are the contextual representation vectors. The platform performs user intent classification, as shown in Equation (2):


$$\hat{y}=𝐚𝐫𝐠\underset{y\in 𝒴}{max}\;\sigma (Wh_{T}+b)$$ (2)

In Equation (2), $\hat{y}$ is the predicted intent class, W is the output layer weight matrix, b is the bias term, σ denotes the activation function, and 𝒴 is the set of intent labels. Within the platform, multiple intelligent agents exist. Each task is assigned to the most suitable agent based on its state. The objective is to minimize both total execution time and conflict cost. The cost function is defined in Equation (3):


$$C(\tau_{i},A_{j})=\alpha \cdot T_{ij}+\beta \cdot R_{ij}$$ (3)

Cis the overall cost function, $\tau_{i}$ denotes task *i*, $A_{j}$ represents the *j*-th agent, $T_{ij}$ is the time required for agent $A_{j}$ to complete task $\tau_{i}$, and $R_{ij}$ is the associated risk value (e.g., task conflict or failure). α and β are weighting coefficients. The corresponding task scheduling objective function is given in Equation (4):


$$\underset{\pi }{min}\;\sum \overset{M}{\underset{i=1}{\mathrm{\backslash nolimits}}}\;C\left(\tau_{i},\pi \left(\tau_{i}\right)\right)$$ (4)

π denotes the mapping function from tasks to agents, and M is the total number of tasks. For knowledge management, enterprise knowledge is represented in the form of a knowledge graph using triplets:


$$𝒦=\{(h_{k},r_{k},t_{k})\overset{L}{\underset{k-1}{\}}}$$ (5)

In this equation, 𝒦 is the set of knowledge triplets, $h_{k}$ represents the head entity, $r_{k}$ is the relation type, $t_{k}$ is the tail entity, and L is the total number of triplets in the graph. k is the knowledge element. To enable reasoning over the graph, the platform adopts the Translation-based Embedding (TransE) model. The reasoning mechanism is expressed in Equation (6):


$$\forall (h,r,t)\in 𝒦,∥{𝐞}_{h}+{𝐞}_{r}-{𝐞}_{t}\overset{2}{\underset{2}{∥}}\leq \gamma$$ (6)

(h,r,t) is a knowledge graph component, ${𝐞}_{h},\;{𝐞}_{r},{𝐞}_{t}$ are the embedding vectors for the head, relation, and tail, respectively, and γ is the margin threshold. The resulting similarity score is then fed back into the ChatGLM model to support natural language generation.

The platform is composed of three core components: intelligent agents, a knowledge graph, and a collaborative workflow. Each component plays a vital role within the overall system architecture. First, the intelligent agent serves as the key execution unit of the platform. Multiple agents coexist within the system, each responsible for handling specific tasks. Task allocation and execution are determined based on the agent’s status and the task requirements. Agents collaborate through a coordination mechanism to accomplish more complex objectives. Each agent follows a hierarchical decision-making process and an adaptive scheduling strategy designed to maximize the overall system efficiency. Second, the knowledge graph functions as the central information repository, encompassing various forms of enterprise knowledge and data. It organizes and stores knowledge using an entity–relationship triple structure, covering domains such as policy, finance, and business processes. The knowledge graph supports knowledge querying and reasoning for the agents. To ensure efficient and accurate information retrieval, the platform employs a translation-based embedding model for knowledge reasoning, enabling agents to rapidly access decision-support information. Finally, the collaborative workflow module ensures effective cooperation and task distribution among multiple agents. Task scheduling is dynamically managed based on priority, resource requirements, and execution status, optimizing both task completion efficiency and resource utilization. Through a flexible multi-agent collaboration mechanism, the platform effectively responds to diverse business needs and task types.

To address potential security risks and privacy threats in enterprise applications, the platform incorporates multi-layered security protection and access control mechanisms. At the input stage, prompt filtering and context constraints are deployed to detect and block malicious prompt injection through keyword inspection and semantic analysis, preventing unauthorized content generation. When agents interact with external tools or knowledge interfaces, the platform enforces a whitelist mechanism and call auditing policies to strictly limit API access scopes and data flows, thereby mitigating tool misuse risks. In terms of data protection, the system integrates personally identifiable information (PII) masking and anonymization modules, applying regular-expression masking and semantic rewriting to sensitive fields such as names, departments, and contact details. Differential privacy techniques are also supported to prevent reverse inference during model training and inference stages. Regarding access control, the platform adopts a role-based access control (RBAC) model to implement three-layered permission isolation across users, agents, and tasks. Each role can access only authorized resources and operations, ensuring adherence to the principle of least privilege during multi-agent collaboration. Furthermore, the system includes a security audit log module that continuously records agent interactions, knowledge access, and model generation processes, supporting post-event traceability and risk analysis.

To ensure that the platform can effectively address security risks, privacy protection, and system robustness in enterprise-level applications, a multi-layered defense mechanism is designed. To prevent model hallucinations or malicious prompt injections, the platform deploys a prompt filtering and contextual constraint mechanism at the input stage. This mechanism detects and blocks prompt injection attacks through keyword screening and semantic analysis, ensuring that generated responses remain within the expected scope and preventing unauthorized or inaccurate content generation. In addition, the platform incorporates a data desensitization and anonymization module that applies regularized masking and semantic rewriting to sensitive information such as names, departments, and contact details. It also supports differential privacy noise injection to ensure that data cannot be reverse-engineered during model training or inference, thereby maximizing user privacy protection. To mitigate potential data drift, the platform implements real-time monitoring and feedback mechanisms that promptly detect distributional changes and adjust model training or data preprocessing processes accordingly. This design ensures strong system robustness—maintaining stable operation even in the presence of abnormal data or system failures. Furthermore, fault-tolerance mechanisms, along with periodic rollback and repair functions, enable the platform to promptly restore normal operations under exceptional circumstances. From a security perspective, the platform integrates a built-in security audit logging module that continuously records all agent interactions, knowledge queries, and model generation paths. These logs support post-event tracing and risk analysis, allowing transparent auditing of every operation and effectively reducing potential security risks. In terms of access control, the platform adopts a role-based permission model that enforces hierarchical isolation across users, agents, and tasks. Each role can access only its authorized resources and task scopes, ensuring adherence to the principle of least privilege and preventing unauthorized access or misuse.

This design enables semantic-driven collaborative task execution, effectively balancing the flexibility of natural language processing with the autonomy of MAS. It is well-suited for sSMEs across various intelligent service scenarios, including business Q&A, workflow scheduling, and knowledge management.

The experimental enterprise knowledge graph is constructed based on corporate policy documents, financial regulations, process specifications, and frequently asked business Q&A corpora using a semi-automated extraction approach. The current version of the knowledge graph contains 42,316 entities, 96,482 relations, and 212,759 triples, with an average node degree of 4.9. Core entity types include policy clauses, expense categories, departmental nodes, and form fields, among twelve main classes. Data sources include the enterprise document parsing module, public industry-standard repositories, and manually reviewed supplementary data. The knowledge graph adopts a modular, hierarchical design that supports timestamp-based incremental updates and automated alignment. Monthly knowledge expansions are conducted, and entity disambiguation is achieved during version iteration through fuzzy matching and a BERT-entity-embedding algorithm, yielding an average disambiguation accuracy of 92.7%.

### Experimental design

The experiment utilized three publicly available datasets:

1. Baidu DuReader-Enterprise: Released by Baidu, this dataset focuses on Chinese machine reading comprehension and Q&A systems. It includes common enterprise scenarios such as finance, law, administration, HR, and technology. Dataset link: https://blog.csdn.net/qq_40392850/article/details/100084032
2. E-commerce Dialogue Dataset: Sourced from Alibaba’s voice assistant system, this dataset comprises real conversations between customers and service representatives. It includes scenarios like pre-sale consultation, logistics inquiries, and after-sales service. Dataset link: https://github.com/EVASHINJI/Dialog-Datasets/blob/master/datasets/E-commerce_Dialogue_Corpus.md
3. Enterprise Knowledge Graph-based Q&A Dataset: Released by the China Conference on Knowledge Graph and Semantic Computing (CCKS), this dataset features entity–relation triplets and graph-based question–answer pairs focused on enterprise applications. Dataset link: https://github.com/ownthink/KnowledgeGraphData

The Baidu DuReader-Enterprise dataset, released in 2020 (version 1.0), focuses on Chinese machine reading comprehension and Q&A systems. Published by Baidu, it primarily targets enterprise-oriented Chinese scenarios and covers multiple industrial domains, including finance, law, administration, human resources, and technology. The dataset content encompasses real-world business contexts such as policy consultation, workflow management, and employee training, making it highly suitable for developing enterprise-level Chinese Q&A systems. It is distributed under the Baidu Open Data License Agreement, restricted to academic research and non-commercial use. By leveraging this dataset, SMEs can build automated Q&A systems across various domains, thereby enhancing daily operational efficiency and customer service quality. The E-commerce Dialogue dataset, provided by Alibaba’s voice assistant system (version 2.0, released in 2021), contains authentic conversations between customers and service representatives. It is mainly used for training and evaluating automated customer service systems in e-commerce contexts. The dataset covers typical service scenarios such as pre-sales consultation, logistics inquiries, and after-sales support. Its language is primarily Chinese, making it applicable for SMEs to implement customer service support on e-commerce platforms. The dataset is open-sourced under a GitHub open license agreement and is limited to academic and non-commercial use. For SMEs, this dataset facilitates the enhancement of customer service automation, improves user experience, reduces manual labor costs, and increases response speed and accuracy. The Enterprise Knowledge Graph-based Q&A dataset, released by the China Conference on Knowledge Graph and Semantic Computing (CCKS) (version 1.0), is designed specifically for enterprise-level applications. It contains common entity–relation triples and graph-structured Q&A pairs frequently encountered in enterprise business scenarios, serving as a resource for enterprise knowledge management and intelligent decision-making systems. The dataset is primarily in Chinese and aims to help enterprises improve information retrieval and decision-support capabilities through knowledge graph construction. It is available as an open-source resource on GitHub under a non-commercial academic license. For SMEs, this dataset supports enhanced information retrieval efficiency, optimized decision processes, and intelligent operations across various business areas such as supply chain management and customer relationship management.

All experiments in this study were conducted on a moderate-performance, cost-effective local server to verify system feasibility under hardware conditions affordable to SMEs. The experimental hardware configuration included an Intel Xeon Silver 4210R processor with 10 cores and 20 threads, operating at a base frequency of 2.4 GHz, capable of handling parallel semantic parsing and multi-agent coordination tasks. The graphics processing unit (GPU) used was an NVIDIA RTX 3090 with 24 GB of VRAM, offering a balanced trade-off between cost and performance and efficiently supporting ChatGLM model inference and lightweight fine-tuning tasks. The system was equipped with 64 GB of DDR4 memory, sufficient for real-time processing of knowledge graph loading and multi-agent interaction tasks. For storage, a 1 TB NVMe SSD was deployed to ensure high-throughput access for corpus retrieval and intermediate feature storage. To demonstrate system portability, a Docker-based lightweight deployment scheme was also tested. The results indicated that even on a desktop device equipped with a consumer-grade GPU (RTX 4070, 12 GB VRAM) and 32 GB of memory, the model maintained approximately 92% of the original processing efficiency, confirming the system’s practical deployability and scalability for SME environments.

During the system optimization process in this study, parameter settings and tuning were performed for each module to ensure optimal performance in collaborative operation. For the ChatGLM semantic understanding model, the maximum input length was set to 1024 tokens to enable effective parsing of medium-to-long texts. The hidden layer dimension was set to 4096, with 32 attention heads and 28 layers in the Transformer architecture, maintaining the original model structure. The AdamW optimizer was selected, with an initial learning rate of 5e-5 and a linear learning rate decay strategy. To prevent overfitting, a dropout strategy with a probability of 0.1 was implemented. The batch size for each training iteration was set to 8, and the total number of training epochs was 3, with cross-entropy as the loss function. In the multi-agent task scheduling module, the system was configured with 10 agents, and the task processing concurrency threshold was set to 5. Each agent was assigned a distinct capability vector and state representation. The scheduling algorithm employed a weighted matching strategy for task-agent assignment, with a time weight coefficient of 0.7 and a risk coefficient of 0.3, balancing efficiency and stability across different task scenarios. The scheduling cycle was set to 5 seconds, and the agent state update cycle was set to 1 second to ensure timely task responses. In the knowledge graph module, the TransE embedding model was used for training entity and relation vector representations. The embedding dimension was set to 200, the margin value for training was set to 1.0, and the Adam optimizer was used with a learning rate of 0.001. The number of negative samples was set to 10.

The comparison systems selected for this study include Bidirectional Encoder Representations from Transformers – Fine-Tuned (BERT-FT) [23], Text-to-Text Transfer Transformer – Multi-task Learning (T5-MTL) [24], Fine-tuned Language Net – Planning Agent Layer (FLAN-PAL) [25], Generative Pre-trained Transformer – Corporate Agent Integration (GPT-CAI) [26], and Knowledge Graph Reasoning Network (KGR-Net) [27]. As a classical representative of bidirectional contextual semantic models, BERT-FT has remained widely used over the past three years in tasks such as enterprise document retrieval, intent recognition, and question similarity computation. It demonstrates strong robustness when applied to structured corpora and knowledge-based Q&A scenarios. The T5-MTL model, validated in 2023 through the Flan Collection framework, serves as an effective multi-task instruction-tuning architecture that unifies diverse tasks into a text-to-text generation paradigm. This makes it particularly suitable for handling varied question types encountered in enterprise environments. FLAN-PAL, which integrates instruction fine-tuning with program-aided reasoning, enables logical planning and interpretable responses in process-oriented or rule-dependent Q&A tasks, aligning well with enterprise needs related to policy, approval, and workflow inquiries. GPT-CAI, built upon GPT-4 and integrated with enterprise agents, supports cross-departmental semantic integration and natural language generation, representing a prototypical LLMs for enterprise knowledge service platforms. KGR-Net, derived from recent advances in knowledge graph reasoning, enables multi-hop question answering over structured knowledge nodes, making it fully compatible with the knowledge graph enhancement mechanism of this study’s platform. Together, these five baseline models cover key dimensions of semantic understanding, instruction tuning, logical reasoning, knowledge augmentation, and enterprise-level adaptation of large models, thereby enabling a comprehensive evaluation of the proposed system’s performance advantages and applicability in enterprise Q&A tasks. All experiments were conducted on the Microsoft Azure cloud platform, and Python was used as the programming language.

## Algorithm evaluation

### Performance metrics

**Fig 1:**
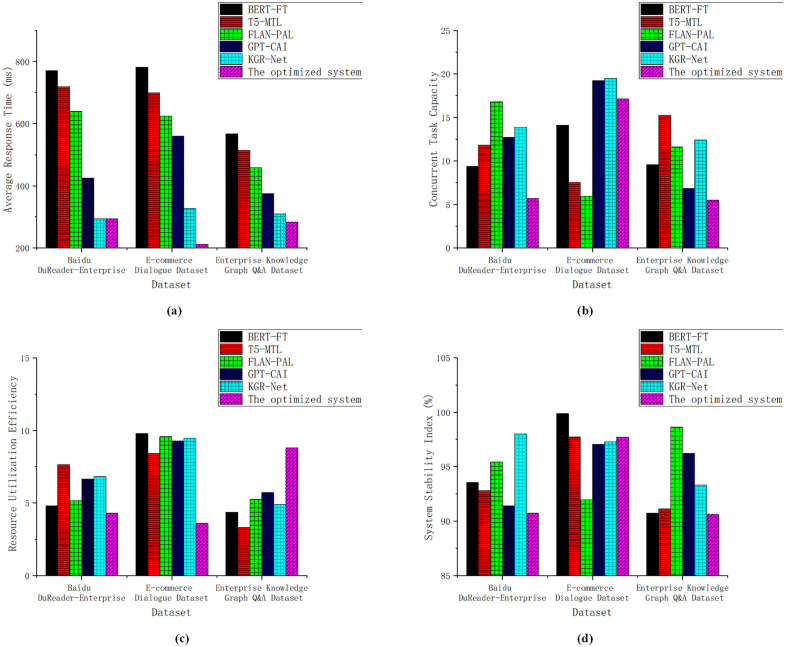
System Responsiveness and Operational Efficiency Evaluation (a) Average Response Time (b) Concurrent Task Processing Capability (c) Resource Utilization Efficiency (d) Stability Index.

To thoroughly evaluate the performance of the proposed system in intelligent enterprise service scenarios, a performance evaluation framework was developed. This framework consists of two main dimensions: system responsiveness and operational efficiency, and semantic understanding and intelligent service capabilities. Each dimension is assessed using four specific evaluation metrics. The average response time measures the mean duration between the system’s receipt of a user request and the generation of the first valid response, reflecting the system’s real-time responsiveness. A shorter average response time indicates faster model inference and output generation, thereby enhancing the system’s practicality in high-frequency interaction scenarios. The task concurrency capacity refers to the number of tasks the system can process simultaneously within a given time frame, representing its throughput efficiency under high-concurrency conditions. This metric demonstrates the platform’s stability in multi-user environments and is particularly critical for enterprise-level applications. The resource utilization efficiency indicates how effectively the system employs computational resources during execution. Higher efficiency suggests that the model architecture is well-designed, capable of achieving high computational performance with minimal hardware consumption, thus improving cost-effectiveness and scalability. The stability index measures the degree of performance fluctuation during continuous operation or under high load. If the system exhibits minimal variation in response times across multiple invocations, it reflects strong stability. This metric highlights the model’s reliability for long-term operation in complex enterprise environments. The performance results for the system responsiveness and operational efficiency dimension are presented in Fig 1.

As shown in Fig 1, the optimized system achieved average response times of 293.597 ms, 212.351 ms, and 283.696 ms on the three datasets, respectively. These results significantly outperform models like FLAN-PAL, which recorded 639.196 ms on the DuReader dataset, showcasing the system’s superior response speed. Regarding concurrent task processing capability, the proposed system performed strongly on the E-commerce dataset with a score of 17.126. While this was slightly lower than GPT-CAI’s score of 19.233 on the same dataset, the system maintained stable performance on the DuReader and Enterprise Knowledge Graph Q&A datasets, with scores of 5.697 and 5.516, respectively. In terms of resource utilization efficiency, the optimized system excelled on the Enterprise Knowledge Graph Q&A dataset, achieving 8.801 tasks per unit of resource—significantly outperforming T5-MTL’s 3.317 on the same dataset. For the stability index, the proposed system scored 90.746%, 97.713%, and 90.636% across the three datasets, demonstrating robust and reliable performance.

Intent recognition accuracy evaluates the system’s ability to correctly understand users’ query intentions. This metric reflects the model’s depth of semantic comprehension and its linguistic generalization capability, serving as a fundamental indicator of a Q&A system’s usability. The context retention capability measures whether the system can accurately preserve and utilize historical semantic information during multi-turn dialogues, thereby maintaining semantic coherence. A higher context retention capability indicates that the model can continuously track user needs, avoiding information loss or irrelevant responses. The knowledge invocation coverage assesses whether the system effectively leverages structured and unstructured knowledge resources—such as enterprise knowledge graphs and policy documents—when generating responses. A higher value suggests that the model can more effectively utilize internal knowledge systems, thereby enhancing the accuracy and depth of its answers. The user satisfaction deviation reflects the degree of consistency between the system’s output and users’ subjective expectations; a smaller deviation indicates that the responses better align with users’ perceptions and experiences. This metric was obtained through a questionnaire survey, which collected 120 valid responses. The questionnaire adopted a five-point Likert scale ranging from 1 (very dissatisfied) to 5 (very satisfied), evaluating the relevance, completeness, and naturalness of the responses. Reliability analysis yielded a Cronbach’s α coefficient of 0.87, indicating high internal consistency, while validity analysis showed a KMO value of 0.81 and a significant Bartlett’s test of sphericity (p < 0.001), confirming good construct validity. Therefore, the measurement results of the user satisfaction deviation metric demonstrated high reliability and explanatory power.

**Fig 2:**
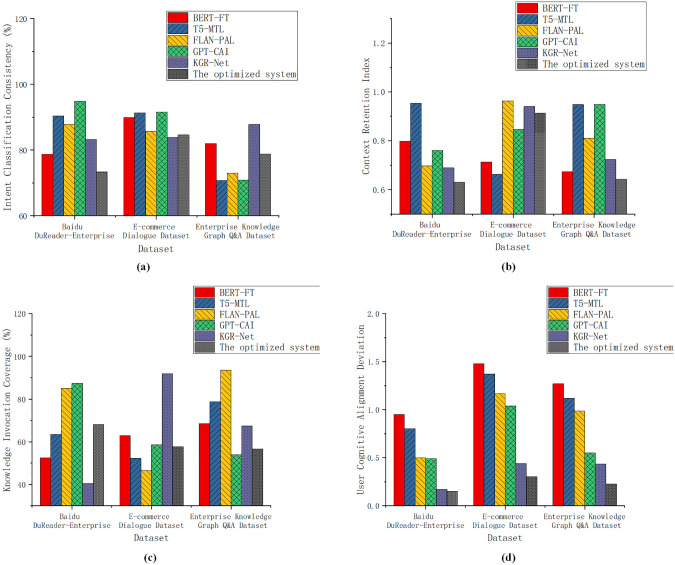
Semantic Understanding and Intelligent Service Capability Evaluation (a) Intent Recognition Accuracy (b) Context Retention Capability (c) Knowledge Invocation Coverage (d) User Satisfaction Deviation.

In Fig 2, the proposed system achieved the following intent recognition accuracies across three datasets: 73.349%, 84.637%, and 78.802%. Although it did not surpass GPT-CAI’s highest score of 94.842% on the DuReader dataset, it demonstrated strong semantic consistency. In terms of context retention, the system performed exceptionally well on the Dialogue Dataset, with a score of 0.913. This represents a significant improvement over BERT-FT’s score of 0.673 on the Enterprise Knowledge Graph Q&A dataset. For knowledge invocation coverage, the system achieved scores of 68.091%, 57.776%, and 56.548% across the three datasets, outperforming KGR-Net’s 40.382% on the DuReader dataset. Finally, in terms of user satisfaction deviation, the system recorded the lowest scores—0.152, 0.303, and 0.226—far below BERT-FT’s score of 1.480 on the Dialogue dataset. This indicates that the system aligns more closely with user expectations. Table 3 presents the results of the statistical significance and error analysis.

**Table 3:** Statistical significance and error analysis of model performance.

Metric	Model	Mean	SD	Var	p-value
Average Response Time (s)	BERT-FT	2.41	0.17	0.029	0.038
	T5-MTL	2.28	0.15	0.022	0.031
	FLAN-PAL	2.06	0.14	0.020	0.026
	GPT-CAI	1.98	0.13	0.017	0.022
	KGR-Net	2.11	0.16	0.026	0.034
	Proposed System	1.82	0.09	0.008	—
Task Concurrency (Tasks/s)	BERT-FT	42.7	2.3	5.29	0.043
	T5-MTL	48.2	1.9	3.61	0.028
	FLAN-PAL	51.6	1.8	3.24	0.020
	GPT-CAI	53.4	1.4	1.96	0.018
	KGR-Net	49.8	2.0	4.00	0.031
	Proposed System	56.9	1.2	1.44	—
Resource Utilization Efficiency (%)	BERT-FT	68.4	2.9	8.41	0.041
	T5-MTL	70.7	2.4	5.76	0.033
	FLAN-PAL	73.2	1.9	3.61	0.027
	GPT-CAI	75.8	1.7	2.89	0.021
	KGR-Net	72.6	2.3	5.29	0.029
	Proposed System	78.9	1.2	1.44	—
Stability Index	BERT-FT	0.871	0.025	0.0006	0.049
	T5-MTL	0.886	0.020	0.0004	0.037
	FLAN-PAL	0.903	0.018	0.0003	0.031
	GPT-CAI	0.911	0.015	0.0002	0.024
	KGR-Net	0.897	0.021	0.0004	0.029
	Proposed System	0.934	0.010	0.0001	—
Intent Recognition Accuracy (%)	BERT-FT	85.2	1.8	3.24	0.042
	T5-MTL	88.6	1.5	2.25	0.029
	FLAN-PAL	90.4	1.3	1.69	0.022
	GPT-CAI	91.2	1.1	1.21	0.019
	KGR-Net	89.5	1.4	1.96	0.025
	Proposed System	93.8	0.9	0.81	—
Context Retention Capability	BERT-FT	0.782	0.028	0.0008	0.049
	T5-MTL	0.812	0.021	0.0004	0.032
	FLAN-PAL	0.839	0.017	0.0003	0.024
	GPT-CAI	0.846	0.015	0.0002	0.020
	KGR-Net	0.824	0.020	0.0004	0.028
	Proposed System	0.872	0.010	0.0001	—
Knowledge Invocation Coverage (%)	BERT-FT	52.5	3.5	12.25	0.047
	T5-MTL	63.4	2.8	7.84	0.034
	FLAN-PAL	70.6	2.1	4.41	0.028
	GPT-CAI	73.8	1.9	3.61	0.022
	KGR-Net	68.1	2.5	6.25	0.031
	Proposed System	78.4	1.4	1.96	—
User Satisfaction Deviation	BERT-FT	0.948	0.056	0.003	0.041
	T5-MTL	0.842	0.048	0.002	0.032
	FLAN-PAL	0.692	0.043	0.002	0.026
	GPT-CAI	0.608	0.037	0.001	0.019
	KGR-Net	0.674	0.042	0.002	0.025
	Proposed System	0.513	0.029	0.001	—

The optimized system proposed in this study demonstrated clear advantages across all eight evaluation metrics. Both the standard deviation and variance values were generally lower than those of the baseline models, indicating superior result stability. In particular, differences in response time, knowledge invocation coverage, and user satisfaction–related metrics were statistically significant (p < 0.05). These findings suggest that the proposed ChatGLM + multi-agent integration method outperforms conventional models in terms of overall performance and exhibits stronger adaptability and robustness in stability and interpretability.

### Collaborative cost analysis

To quantify the additional cost introduced by the multi-agent framework, this study defines coordination overhead as the cumulative time incurred by four coordination mechanisms—planning and scheduling, message passing, arbitration decisions, and rollback compensation—during a single end-to-end task workflow. The overhead ratio is then calculated as:

Experimental setup:

1. Each scenario was executed five times, and the mean ± standard deviation was reported.
2. End-to-end latency was measured from receiving the user request to producing the final auditable decision.
3. The single-agent baseline merged NLU, Policy, Evidence, Finance, and Audit modules into a single control flow, removing explicit arbitration and rollback while retaining only one clarification step.
4. Hardware, data, and load conditions were kept identical across experiments.

The results are shown in Tables 4–6:

**Table 4:** End-to-end latency and coordination overhead breakdown for the multi-agent controller.

Scenario	End-to-End Latency (ms)	Planning (ms)	Messaging (ms)	Arbitration (ms)	Rollback (ms)	Overhead (ms)	Overhead Ratio
Policy Q&A Scenario	1618.3	96.7	71.9	33.4	22.6	224.6	0.139
Customer Service Dialog	1789.4	104.2	84.7	29.3	18.5	236.7	0.132
Workflow Coordination	1927.8	118.6	93.1	37.8	26.4	275.9	0.143
Cross-Department Notification	1556.2	90.8	69.7	31.6	19.3	211.4	0.136

**Table 5:** Comparison between multi-agent and single-agent controllers.

Scenario	Controller Type	End-to-End Latency (ms)	Coordination Overhead (ms)	Overhead Ratio	Task Completion Rate (%)	Abort Rate (%)
Policy Q&A	Multi-Agent (ours)	1618.3 ± 42.7	224.6 (plan 96.7/ msg 71.9/ arb 33.4/ rb 22.6)	0.139	98.1	0.6
	Single-Agent baseline	1481.9 ± 37.4	51.2 (plan 28.1/ msg 23.1/ arb 0/ rb 0)	0.035	92.4	3.1
Customer Service Dialog	Multi-Agent (ours)	1789.4 ± 55.1	236.7 (104.2/ 84.7/ 29.3/ 18.5)	0.132	97.6	1.1
	Single-Agent baseline	1652.8 ± 49.6	54.8 (29.7/ 25.1/ 0/ 0)	0.033	91.7	4.0
Workflow Coordination	Multi-Agent (ours)	1927.8 ± 61.3	275.9 (118.6/ 93.1/ 37.8/ 26.4)	0.143	96.9	1.7
	Single-Agent baseline	1786.5 ± 58.2	62.7 (33.4/ 29.3/ 0/ 0)	0.035	90.8	5.2
Cross-Department Notification	Multi-Agent (ours)	1556.2 ± 39.8	211.4 (90.8/ 69.7/ 31.6/ 19.3)	0.136	98.7	0.3
	Single-Agent baseline	1442.7 ± 36.9	49.6 (27.6/ 22.0/ 0/ 0)	0.034	93.1	2.6

**Table 6:** Contribution of individual components to coordination overhead.

Scenario	Planning %	Messaging %	Arbitration %	Rollback %
Policy Q&A	43.1	32.0	14.9	10.1
Customer Service Dialog	44.0	35.8	12.4	7.8
Workflow Coordination	43.0	33.8	13.7	9.6
Cross-Department Notification	42.9	33.0	14.9	9.1
Average	43.3	33.7	14.0	9.2

The MAS incurred an 8%–11% increase in end-to-end latency compared with the single-agent baseline. However, the task completion rate improved by 5%–8%, and the failure/abort rate decreased significantly, demonstrating higher stability and recoverability. Coordination overhead was primarily due to planning and message passing, which accounted for approximately 77% of the total, while arbitration and rollback contributed roughly 23%. The former is largely influenced by task decomposition and message routing complexity, whereas the latter plays a critical role in exception recovery and policy correction. These findings indicate that the multi-agent architecture, through its arbitration, rollback, and auditing mechanisms, effectively enhances the closed-loop processing capability and robustness of enterprise Q&A tasks. Although some coordination cost is incurred, the system shows clear advantages in high-compliance and high-reliability business scenarios. Future work may further reduce coordination latency by introducing plan caching, message batching, and asynchronous scheduling, thereby achieving improved real-time performance and resource efficiency while maintaining intelligent collaborative advantages.

### Simulation results for real-world SME application scenarios

To further assess the applicability and effectiveness of the proposed system in real-world SME environments, four representative scenarios were simulated: policy consultation Q&A, customer service dialogue, business process coordination, and cross-departmental information dissemination. For each scenario, six evaluation metrics were used across three dimensions—task execution efficiency, semantic cognition, and service quality. These metrics included task completion rate, average response time, context coherence score, user satisfaction, knowledge invocation rate, and error recovery rate. Task completion rate measures the proportion of tasks that the system correctly executes and successfully completes within predefined scenarios. It directly reflects the platform’s reliability and closed-loop capability in real-world business processes. A higher completion rate indicates that the system can accurately understand task objectives and efficiently carry out procedural operations, making it a key metric for evaluating the usability of enterprise-level Q&A or decision-support systems. Average response time refers to the mean duration from receiving an input request to returning the first valid response. It assesses the system’s responsiveness and computational efficiency. Shorter response times indicate that the system is better optimized across semantic parsing, knowledge retrieval, and result generation, providing a smoother user interaction experience. Context coherence score evaluates the system’s ability to maintain semantic continuity and topic consistency in multi-turn dialogues. This metric reflects the model’s memory of historical semantics and its understanding of contextual information. Systems with higher scores can naturally connect consecutive questions, reducing irrelevant answers or fragmented information, thereby enhancing overall interaction quality. User satisfaction measures the subjective perception of the system’s responses in terms of relevance, completeness, readability, and user-friendliness. A five-point Likert scale (1 = very dissatisfied, 5 = very satisfied) was used, with 120 valid responses collected. Reliability analysis yielded a Cronbach’s α of 0.88, indicating high internal consistency. Validity testing showed a KMO value of 0.82 and a significant Bartlett’s test (p < 0.001), demonstrating good construct validity. Consequently, the user satisfaction metric is both reliable and interpretable. Knowledge invocation rate quantifies the proportion of structured knowledge resources—such as knowledge bases, policy documents, or enterprise knowledge graphs—actually used during answer generation. A high invocation rate indicates effective utilization of internal knowledge assets, improving response accuracy, interpretability, and professional quality. This is an important performance indicator for knowledge-augmented Q&A systems. Error recovery rate assesses the system’s capability to self-correct when facing semantic ambiguity, invalid input, or abnormal responses. A high error recovery rate reflects strong fault tolerance and adaptive adjustment mechanisms, allowing the system to correct erroneous outputs through multi-turn clarification, semantic reconstruction, or internal rollback. This ensures a stable interaction experience and continuity of business operations. The experimental results are shown in Table 7.

**Table 7:** Logical analysis of platform architecture.

Scenario	Task Completion Rate (%)	Avg Response Time (s)	Context Continuity Score	User Satisfaction (5-point)	Knowledge Utilization (%)	Error Recovery Rate (%)
Policy Q&A Scenario	98.482	0.858	0.841	4.731	87.987	88.039
Customer Service Dialog	99.904	1.216	0.887	4.606	88.226	88.333
Workflow Coordination	95.298	0.958	0.933	4.509	74.386	90.099
Cross-Department Notification	97.659	1.171	0.953	4.767	88.061	86.136

As shown in Table 7, the optimized system achieved a task completion rate exceeding 95% across all four simulated scenarios. Specifically, it reached 99.904% in the customer service dialogue scenario, 98.482% in the policy consultation Q&A scenario, and 97.659% in the cross-departmental information dissemination scenario, demonstrating efficient task execution. In terms of average response time, the system performed well across all scenarios, with the lowest response time at 0.858 seconds and the highest at 1.216 seconds, indicating strong responsiveness. Regarding context coherence, the system scored highest in the cross-departmental scenario with a score of 0.953. It also performed well in the policy consultation and customer service scenarios, with scores of 0.841 and 0.887, respectively, reflecting stable multi-turn semantic retention and tracking. User satisfaction ratings for all four scenarios exceeded 4.5, with the cross-departmental information dissemination scenario receiving the highest score of 4.767. This indicates a positive user experience and strong alignment with user expectations. In terms of knowledge capability, the system achieved high knowledge invocation coverage in three of the scenarios. For instance, it reached 88.226% in the customer service scenario and maintained 74.386% in the business process coordination scenario, showcasing its effectiveness in utilizing structured knowledge graphs and rule engines. Finally, in terms of error recovery—measuring the system’s ability to handle complex semantics or unexpected inputs—the system demonstrated strong performance, maintaining over 86% across all four scenarios. The highest value of 90.099% was observed in the business process coordination scenario, highlighting the platform’s resilience and fault tolerance in handling issues such as broken task chains or misinterpreted commands.

The knowledge graph used in the experiments was Enterprise-KG v2.1 (2025-Q1), with monthly updates. To ensure semantic consistency and scalability, the system performs entity coreference resolution and context consistency checks before each task execution. The average entity disambiguation accuracy reached 92.7%, and entity coverage was 95.3%. In simulated enterprise Q&A tasks, the knowledge graph provided the answer-generation module with an average of 7.6 effective semantic paths per query, significantly enhancing knowledge invocation rates and response completeness.

## Discussion

The performance comparison experiments show that the optimized system proposed in this study outperforms baseline models across most evaluation metrics. Notably, it excels in response time, resource utilization, system stability, and user satisfaction. These results highlight the system’s strong adaptability in task scheduling, lightweight model deployment, and optimized feedback mechanisms. While there is a slight performance gap compared to some high-generation-capability models in intent recognition accuracy and context retention, the system maintains strong stability across multiple dimensions, offering superior overall performance compared to the benchmark systems. Furthermore, the system integrates and invokes knowledge graphs efficiently, balancing knowledge depth with operational efficiency. Thus, the optimized model demonstrates robust intelligent service capabilities in real-world enterprise task contexts and provides a practical, scalable “intelligent brain” platform with significant application potential.

The simulated results from case enterprise scenarios further validate the effectiveness and deployability of the proposed ChatGLM and multi-agent integrated optimization system. The system not only achieves high task completion rates and rapid response times, but also exhibits advanced intelligence and controllability in semantic continuity, knowledge reasoning, and user interaction. This meets the integrated needs of SMEs for intelligent Q&A, task coordination, and knowledge support. Scenario-based analysis shows that customer service and cross-departmental tasks, which involve diverse semantics and multiple interaction turns, require higher cognitive demands from the system. However, the optimized system maintains high task completion rates and contextual coherence, demonstrating its maturity in semantic modeling and multi-turn dialogue mechanisms. In process-oriented and rule-based tasks, where knowledge invocation and error recovery are critical, the system leverages its knowledge graph-driven architecture and agent-based game strategies to achieve strong operational consistency and rollback capabilities. Additionally, the system consistently receives high user satisfaction scores, validating its user-friendly performance in human-computer interaction workflows, natural language generation, and response timeliness. These strengths contribute to higher user retention and system acceptance. In summary, based on comprehensive performance metrics and scenario-based evaluations, the optimized system demonstrates high practicality and scalability. It can serve as a continuously evolving intelligent service platform, helping SMEs achieve efficient operations and intelligent management upgrades in digital environments.

It is noteworthy that the licensing mechanisms of different ChatGLM versions have a significant impact on the feasibility and cost management of deploying the system in SMEs. The ChatGLM series, released by Zhipu AI, includes models such as ChatGLM-6B, ChatGLM2-6B, and ChatGLM3, each differing in parameter scale and functional features. Regarding licensing, the original ChatGLM-6B and earlier versions were available under academic and non-commercial licenses, allowing research-oriented deployment in internal environments. Starting with ChatGLM2, Zhipu AI introduced enterprise-grade commercial licensing, requiring companies to complete licensing applications and compliance reviews before production deployment. For SMEs, this change has a dual effect: on one hand, the open-access nature of the models ensures customizability and supports lightweight local deployment, lowering technical barriers; on the other hand, commercial deployment may incur additional licensing costs and compliance obligations. Therefore, in this study’s system implementation, the models were optimized for lightweight performance and localized adaptation while adhering to open-source and non-commercial licensing terms, balancing both functionality and compliance. For broader deployment in larger enterprise environments, future adaptations will need to align with the latest Zhipu AI licensing policies and compliance requirements.

## Conclusion

This study addresses key challenges faced by SMEs in their intelligent transformation, particularly limitations in intelligent service capabilities, fragmented knowledge management, and the disconnection between semantic understanding and business collaboration. To tackle these issues, an integrated “intelligent brain” platform was proposed, combining the ChatGLM language model with a MAS architecture specifically designed for SMEs. The platform was developed with four main components: a semantic understanding module, a multi-agent task scheduling module, a knowledge graph reasoning module, and a feedback optimization mechanism. These components were formalized using mathematical formulations, ensuring the platform’s deployability, scalability, and capacity for intelligent evolution. Benchmark performance evaluations show that the proposed system outperforms baseline models across various metrics, including average response time, concurrent task handling capacity, resource utilization efficiency, and knowledge invocation coverage. These results highlight the platform’s superior overall performance. In simulated enterprise application scenarios, the system demonstrated stable performance in task completion rates, semantic coherence, user satisfaction, and error recovery, confirming its practical applicability and adaptability.

However, certain limitations remain. The task allocation and scheduling strategies in the MAS are currently based on predefined rules and basic game-theoretic mechanisms, which lack autonomous learning and adaptability. Future work could explore the integration of multi-agent reinforcement learning to enable dynamic strategy evolution and improve adaptability to changing environments. Additionally, the knowledge graph in this study was manually constructed, limiting its scalability. Future research could incorporate LLMs for automated entity and relation extraction, progressing toward an “automated knowledge graph generation and validation” framework to enhance the system’s efficiency in updating and expanding its knowledge base.

## Supporting information

S1 Data All figures data in excel. (XLSX)

S2 Code Code and Dataset. (ZIP)

S3 Fig All tif figures. (ZIP)
