# Supplementary material for: Building an intelligent brain platform for small and medium-sized enterprises using ChatGLM and Multi-Agent Systems
Source: PLoS One. 2026 Mar 27;21(3):e0340964. doi: 10.1371/journal.pone.0340964 (PMC13028509; doi:10.1371/journal.pone.0340964)
Supplement: S2 Code — (ZIP) [file pone.0340964.s002.zip › Code and Dataset/Code Description.docx]

**I. Global Logging and Model Initialization**

1. **Logging Initialization**
   - Set the logging level to INFO and define the output format to facilitate tracking of key events during system operation (such as model invocation, task dispatching, feedback recording, etc.).
2. **ChatGLM Model Class**
   - ChatGLM.__init__(): Loads the fine-tuned model from the specified path;
   - parse(text): Simulates intent recognition and entity extraction, returning a dictionary format, e.g., intent as "QUERY_POLICY";
   - generate_response(context): Generates natural language responses, simulating platform replies;
   - fine_tune(data): Performs model fine-tuning, triggered when user feedback indicates poor response quality.
3. **KnowledgeGraph Class**
   - query(entity): Simulates entity queries and returns results from the graph;
   - add_entity(entity, info): Adds new knowledge nodes to the graph to support enterprise knowledge structure expansion.

**II. Agent Framework Module**

1. **Agent Class**
   - Each agent has a unique ID (e.g., A001), a role (e.g., "KG_QUERY"), and a set of skills (e.g., "finance");
   - assign_task(task): Assigns a task and switches to a busy state;
   - execute_task(): Simulates task execution (with delay) and returns to idle state upon completion;
   - get_status(): Returns the current status, serving as a reference for the scheduler.

**III. Session and Task Management Module**

1. **SessionManager Class**
   - create_session(user_id): Generates a unique session (UUID) based on the user ID to identify each interaction;
   - get_session(user_id): Retrieves an existing session ID or creates a new one if none exists, ensuring interaction continuity.
2. **TaskManager Class**
   - decompose_intent(intent_data): Decomposes complex tasks into multiple subtasks (e.g., querying multiple entities) based on semantic parsing results, and returns a task queue.

**IV. Task Scheduling and Execution Monitoring Module**

1. **Dispatcher Class**
   - dispatch(task): Searches for all idle agents with matching roles and assigns the task to the first eligible one;
   - If no idle agents are available, it issues a warning: “Task will be queued,” indicating the need for platform scaling or queueing.
2. **MetricsLogger Class**
   - log_event(session_id, event_type, detail): Logs every event related to a user session, including user input and task dispatching;
   - Stores event information in dictionary format with timestamps for subsequent behavior analysis and system optimization.

**V. Feedback Collection and Adaptive Learning Module**

1. **FeedbackEngine Class**
   - record_feedback(session_id, task_result, score): Records the user feedback score and output result for each session round;
   - evaluate(): Extracts task results with scores below 3 from the feedback data to prepare training samples for fine-tuning.

**VI. Core Platform Controller (EnterpriseBrainPlatform Class)**

1. **Constructor __init__()**

- Initializes subsystem components, including the model, knowledge graph, session manager, task manager, logger, feedback system, and agent collection;
- Initializes agents with three roles:
  - A001: Knowledge graph querying (KG_QUERY)
  - A002: General text response (CHAT_RESPONSE)
  - A003: Workflow processing (WORKFLOW_CALL)

1. **process_user_input(user_id, text)**
   - Retrieves or creates a session ID;
   - Logs the user input;
   - Invokes the model for semantic parsing;
   - Decomposes the intent into subtasks;
   - Dispatches tasks via the scheduler and logs each dispatch event.
2. **collect_user_feedback(session_id, rating)**
   - Records the user's rating of the output for later model performance evaluation.
3. **retrain_model_if_needed()**
   - Invokes the feedback evaluation method;
   - If low-rated tasks are found, triggers model fine-tuning using those failed samples to improve performance.

**VII. Main Program Entry (Main Function)**

1. **User Input Simulation**
   - Simulates three users sequentially asking questions, such as reimbursement policy, leave process, and expense claims;
   - Each input is handled by calling process_user_input().
2. **User Feedback Simulation**
   - Simulates feedback scoring for each session using collect_user_feedback();
   - Random scores are generated to simulate real user experience.
3. **Dynamic Model Training**
   - Calls retrain_model_if_needed() to determine whether retraining is necessary;
   - If poor-quality dialogues are identified, the ChatGLM fine-tuning process is triggered.
