# Supplementary material for: Building an intelligent brain platform for small and medium-sized enterprises using ChatGLM and Multi-Agent Systems
Source: PLoS One. 2026 Mar 27;21(3):e0340964. doi: 10.1371/journal.pone.0340964 (PMC13028509; doi:10.1371/journal.pone.0340964)
Supplement: S2 Code — (ZIP) [file pone.0340964.s002.zip › Code and Dataset/Dataset Description.docx]

**Baidu DuReader-Enterprise Dataset Overview**

**1. Dataset Introduction**

Baidu DuReader-Enterprise is an enterprise-oriented question answering dataset developed by Baidu, extended from the original DuReader dataset. Its goal is to build a high-quality Chinese QA resource tailored for real-world work environments and focused on domain-specific issues. This dataset aims to support intelligent semantic needs in areas such as knowledge management, intelligent customer service, and process consulting within enterprise contexts.

**2. Background and Objectives**

As intelligent QA systems become more common in corporate document search, employee services, and knowledge-based queries, traditional QA datasets—such as SQuAD and the original DuReader—often fall short. They struggle to capture the necessary context, linguistic style, and semantic complexity found in real enterprise environments. DuReader-Enterprise was created to bridge this gap. It focuses on key business areas within medium-to-large enterprises, including government affairs, finance, human resources, legal compliance, and reimbursement policies. The dataset features corpus content designed to reflect real-world scenarios, improving both the professionalism and practical utility of QA systems.

**3. Data Scale and Sources**

- **Total QA Pairs**: Over 50,000 real-world, business-related question-answer pairs;
- **Primary Sources**: Enterprise employee knowledge bases, government and enterprise policy documents, internal business SOPs, HR/Finance FAQs compilations;
- **Covered Domains**: Human resource management, financial regulations, business workflows, corporate culture, administrative affairs, compliance consulting, etc.;
- **Construction Methods**: Manually designed questions + collected user query corpora + expert knowledge document generation.

**4. Data Structure and Format**

DuReader-Enterprise is typically formatted in JSON, with the following structure:

{

"question": "How can an employee apply for overtime reimbursement?",

"paragraphs": [

{

"context": "According to the Overtime Management Policy, employees must complete the 'Overtime Approval Form' within three days after the overtime ends and submit it to the HR department after obtaining the supervisor's signature.",

"answers": [

{

"text": "Complete the 'Overtime Approval Form' and submit it to the HR department",

"start_position": 18

}

]

}

]

}

- question: A natural language query from the user;
- context: A segment from enterprise documents (typically rules, process descriptions, internal notices, etc.);
- answers: Reference answers annotated manually or semi-automatically, along with their positions in the context.

**5. Annotation Strategy and Features**

- **Multi-turn QA Support**: Some samples are designed as part of a multi-turn dialogue chain (e.g., a follow-up question like “Does it need supervisor approval?”);
- **Context Nesting**: Some contexts include not just narrative text, but also structured fields such as “Process Steps” or “Important Notes”;
- **Answer Diversity**: Certain questions may have multiple valid answers (e.g., different document versions or process paths);
- **Intent Classification Adaptation**: In addition to the QA structure, intent labels are included (e.g., policy explanation, process inquiry, permission clarification), facilitating intent recognition training in models.

**6. Enterprise Application Advantages and Practical Value**

- **High Semantic Compatibility**: The questions and answers reflect the language style typically used within enterprises, closely aligning with real HR, finance, and administrative expressions;
- **Strong Domain Adaptability**: Covers common business processes and employee inquiries in small-to-medium-sized enterprises, making it easy to fine-tune models for enterprise-specific QA applications;
- **Support for Multi-task Training**: Suitable for a variety of NLP subtasks including intent recognition, question-answer matching, paragraph ranking, and answer extraction;
- **Good Scalability**: Enterprises can extend the dataset by incorporating their own document formats into the context field, allowing for customized sample augmentation and training.

**7. Key Differences from the General DuReader Version**

| **Feature** | **General DuReader** | **DuReader-Enterprise** |
| --- | --- | --- |
| **Question Type** | Open-domain questions from news and encyclopedias | Closed-domain questions related to enterprise operations |
| **Data Source** | User queries from search engines + encyclopedia paragraphs | Internal enterprise documents + policy and regulation materials |
| **Answer Characteristics** | Generalized answers with broader information scope | Precise business expressions with focus on procedures and regulatory references |
| **Language Style** | Generic, public-facing QA language | Enterprise-specific terminology, regulatory language, and administrative expressions |
| **Application Scenarios** | General-purpose NLP QA tasks | Internal enterprise QA systems, intelligent customer service, knowledge recommendation, etc. |

**E-commerce Dialogue Dataset Overview**

**1. Dataset Introduction**

The E-commerce Dialogue Dataset is a multi-turn human-computer dialogue dataset tailored for e-commerce scenarios, developed by research teams including Alibaba DAMO Academy. It is designed to support intelligent customer service systems in the e-commerce domain with high-quality dialogue corpora. Widely used in tasks such as multi-turn dialogue modeling, intent recognition, dialogue state tracking, and response generation, this dataset is one of the most representative resources for Chinese dialogue semantics research.

**2. Background and Core Objectives**

In e-commerce services, users often interact with online customer service agents for pre-sale and post-sale inquiries in multiple rounds of conversation. These dialogues are highly task-oriented, emotionally expressive, and heavily context-dependent. To train intelligent customer service systems capable of understanding user needs, responding promptly, and tracking context over time, the E-commerce Dialogue Dataset systematically collects, cleans, and annotates real-world service dialogues.

The dataset aims to address the following challenges:

- Lack of high-quality dialogue corpora in e-commerce customer service contexts;
- Insufficient domain knowledge comprehension by general-purpose dialogue models;
- Modeling difficulties caused by common characteristics in e-commerce conversations, such as diverse intents, contextual jumps, and unstructured expressions.

**3. Data Sources and Scale**

- **Sources**: Real chat logs from platforms such as Taobao and Tmall;
- **Number of Dialogues**: Over 700,000 multi-turn dialogue records;
- **Turns per Dialogue**: Most dialogues contain 3–10 turns, with some extending up to 30 turns;
- **Format**: Text-based dialogues between users (Buyer) and customer service agents (Agent);
- **Anonymization**: All privacy-related, brand-specific, and sensitive information has been anonymized or abstracted.

**4. Data Structure and Annotation Format**

The dataset is organized in JSON format. Each dialogue entry typically includes the following fields:

json

{

"dialogue_id": "123456",

"turns": [

{"role": "Buyer", "text": "Hi, does this clothing item come in large size?"},

{"role": "Agent", "text": "Hello, yes it does. May I ask which color you're interested in?"},

{"role": "Buyer", "text": "Blue, thank you."}

],

"intent_labels": ["Inquire Size", "Product Recommendation", "Confirm Color"]

}

- turns: A list of dialogue exchanges between user and agent;
- role: Indicates the speaker (Buyer or Agent);
- text: The textual content of each utterance;
- intent_labels *(optional)*: Annotated user intents for each turn, such as "Product Inquiry", "Logistics Query", "After-sales Request", etc.

**Dialogue Style and Linguistic Features**

- **Dialogue acts**: Annotated dialogue act labels indicating the function of each utterance (e.g., request, inform, confirm);
- **Slot values**: Slot filling results such as product_category = dress;
- **Emotion tag**: Trends of user emotional changes throughout the dialogue;
- **Action suggestion**: Recommended response strategies or actions.

**5. Linguistic Characteristics**

- **Highly colloquial expressions**: Natural and realistic conversations containing filler words, ellipses, and vague phrasing;
- **Strong contextual dependency**: Includes co-reference (e.g., “this one”, “that item”) and ellipses (e.g., “Got it?”);
- **Significant emotional shifts**: Notably observed during complaint handling, delayed delivery, and return/exchange requests;
- **Prevalence of unstructured inputs**: Many user utterances do not follow standard grammar, challenging the system’s robustness and comprehension.

**6. Application Tasks and Research Value**

This dataset is widely used for the following tasks:

| **Task** | **Description** |
| --- | --- |
| Multi-turn Dialogue Modeling | Train models capable of tracking dialogue state and understanding context. |
| Intent Recognition | Classify the task intent of each user utterance in the dialogue flow. |
| Dialogue Policy Learning | Learn how a customer service agent should plan response strategies (e.g., guide, clarify, escalate, close). |
| Automatic Response Generation | Generate natural, relevant, and personalized agent responses based on dialogue history. |
| Emotion Recognition and Mitigation | Detect user emotional tendencies and generate empathetic, logically calming responses. |

**7. Significance in Enterprise Dialogue Systems**

For building intelligent semantic platforms or “Enterprise Brain” systems, the E-commerce Dialogue Dataset offers:

- **Rich, real-world interaction samples**: Enabling the training of dialogue agents with both contextual awareness and task responsiveness;
- **End-to-end coverage of inquiry–recommendation–complaint processes**: Providing typical business flows for intent classification and module orchestration;
- **Integration of emotional and UX management scenarios**: Especially beneficial for service-oriented SMEs seeking to build highly interactive semantic service interfaces.

**Enterprise Knowledge Graph-based QA Dataset Overview**

**1. Introduction**

The Enterprise Knowledge Graph-based QA Dataset (Enterprise KG-QA Dataset) is specifically designed for enterprise-level business knowledge management and intelligent question answering systems. Based on knowledge graph triples, the dataset structurally represents enterprise policies, processes, systems, products, and organizational information, and links these with corresponding QA pairs. The goal is to enable large language models or QA systems to reason, retrieve, and respond to natural language queries using structured knowledge.

**2. Background and Objectives**

As enterprise knowledge management evolves from unstructured documents to structured knowledge graphs, KG-QA systems have become a core capability in intelligent enterprise service platforms. Unlike open-domain QA or FAQ matching, graph-based QA offers more stable, interpretable, and standardized semantic outputs. This dataset was constructed to enhance a system's capabilities in reasoning, structured knowledge recognition, and domain-specific semantic mapping.

**3. Data Sources and Knowledge Domains**

**• Sources:**

- Enterprise regulations and policy documents (e.g., employee handbooks, salary systems, attendance policies);
- Business process documentation (e.g., procurement, reimbursement, budgeting, travel approvals);
- Structured internal data such as product info, org charts, HR records, and project categories;
- Some QA pairs are synthetically generated using template + entity combinations.

**• Covered Domains:**

- Human Resources (HR), Finance, Administration, Legal, Customer Management, Product Management;
- Common knowledge topics include role permissions, business conditions, operational procedures, policy content, and responsibility division.

**4. Data Format and Structure**

The dataset consists of two main components:

**A. Knowledge Graph Triples**
Format: (subject, predicate, object)
Examples:

- ("Reimbursement Application", "requires submission of", "original invoice")
- ("Onboarding Process", "includes step", "new employee training")
- ("Department Supervisor", "has approval authority over", "overtime request")

**B. QA Pairs**

Format (JSON):

json

{

"question": "What materials are required for reimbursement?",

"triple_links": [

["Reimbursement Application", "requires submission of", "original invoice"],

["Reimbursement Application", "requires submission of", "approval form"]

],

"answer": "You need to submit the original invoice and the approval form."

}

- question: Natural language user query;
- triple_links: References to supporting knowledge triples from the graph;
- answer: A standardized, accurate natural language response with traceable evidence.

**5. Dataset Features and Annotation Scheme**

- **Standardized Triples**: All entities and relations follow an enterprise terminology schema to avoid ambiguity or redundancy;
- **Question Diversity**: Covers a variety of query types such as factual, conditional, and procedural questions;
- **Explicit Reasoning Paths**: Each answer is traceable to one or more graph paths, supporting multi-hop reasoning;
- **High Answer Precision**: Answers are crafted to be clear, executable, and logically complete, fitting enterprise application needs;
- **Intent Annotation Support**: Some versions include semantic intent tags such as “policy explanation” or “role responsibility” for enhanced model training.

**6. Sample Data Illustration**

| **Question** | **Answer** | **Associated Triples** |
| --- | --- | --- |
| "What are the steps in the onboarding process?" | "They include document completion, training, and access requests." | [("Onboarding Process", "includes step", "Document Completion"), ("Onboarding Process", "includes step", "Training")] |
| "Who can approve overtime requests?" | "The department supervisor has approval authority." | [("Department Supervisor", "has approval authority over", "Overtime Request")] |
| "What is the first step in the procurement process?" | "Filling out the procurement application form." | [("Procurement Process", "starting step", "Fill out procurement application form")] |

**7. Applicable Tasks and Research Directions**

| **Task Direction** | **Description** |
| --- | --- |
| **KG-QA Model Training** | Train QA systems capable of understanding, reasoning, and answering using knowledge graphs. |
| **Multi-hop Question Answering** | Enable reasoning across multiple triples to form complex answers, enhancing logical comprehension. |
| **Question Rewriting / Entity Linking** | Align natural language questions with graph entities, supporting domain-specific semantic mapping. |
| **Enterprise Semantic Search** | Enable hybrid semantic + structural search to improve interaction quality and answer precision in enterprise knowledge systems. |

**8. Enterprise Applications and Value Proposition**

- **Enhanced Domain Knowledge Support**: Compared to generic QA models, KG-QA offers more **accurate and regulation-compliant** structured responses.
- **Improved System Explainability**: All answers are traceable to knowledge graph triples, **boosting credibility and transparency**.
- **Ease of System Maintenance and Upgrades**: Updates can be made by **modifying triples** rather than retraining the entire model.
- **Compatibility with LLMs (e.g., ChatGLM)**: Well-suited for **prompt-based inference** and graph-augmented large model training.
- **Ideal for Building Hybrid Enterprise Intelligence Platforms**: Enables **fusion of documents and structured knowledge**, forming a dynamic enterprise "brain".
