# Supplementary figures and images for: Building an intelligent brain platform for small and medium-sized enterprises using ChatGLM and Multi-Agent Systems
Source: PLoS One. 2026 Mar 27;21(3):e0340964. doi: 10.1371/journal.pone.0340964 (PMC13028509; doi:10.1371/journal.pone.0340964)

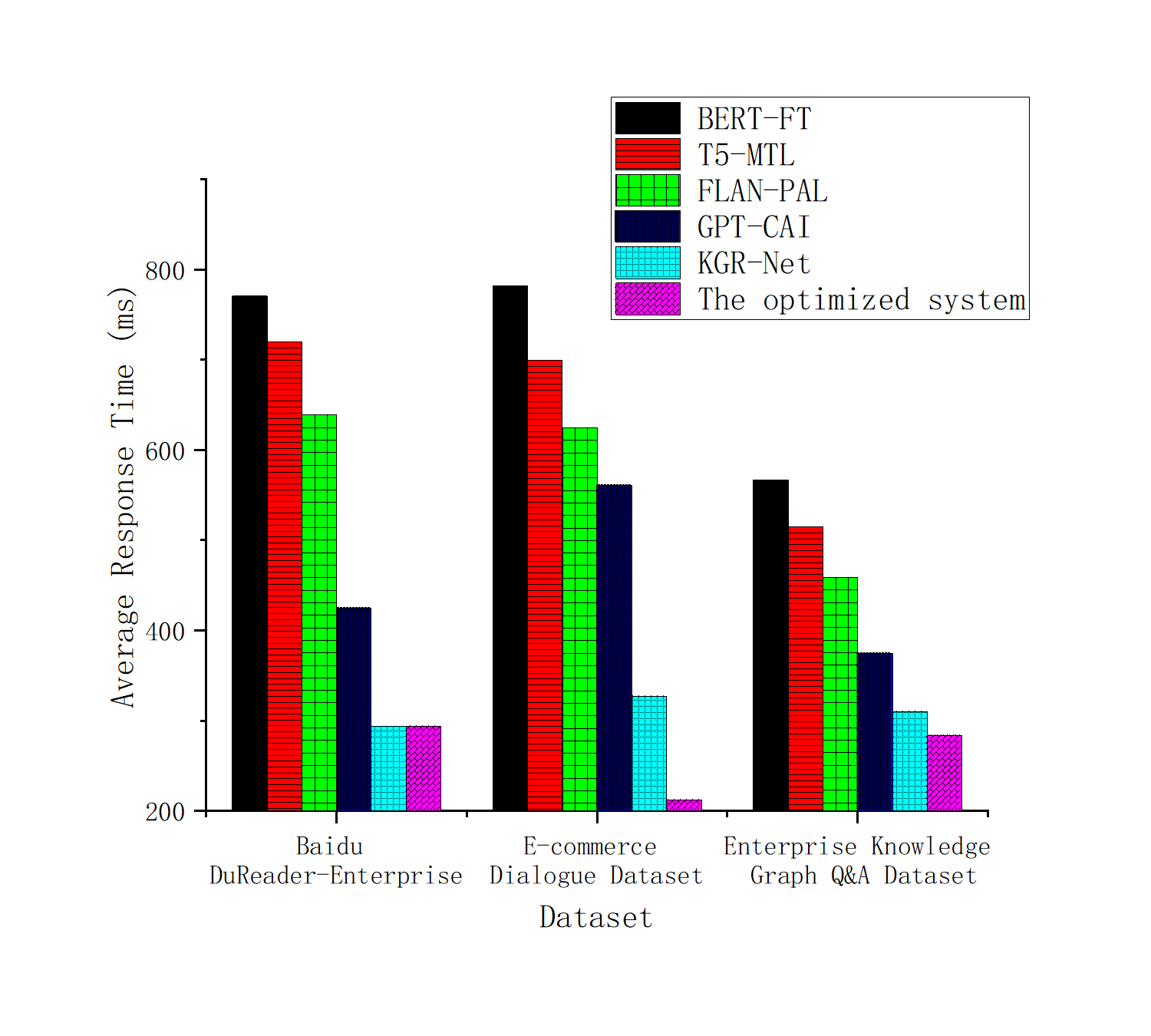

Supplement: S3 Fig — (ZIP) [file pone.0340964.s003.zip › Fig/Fig1(a).tif]

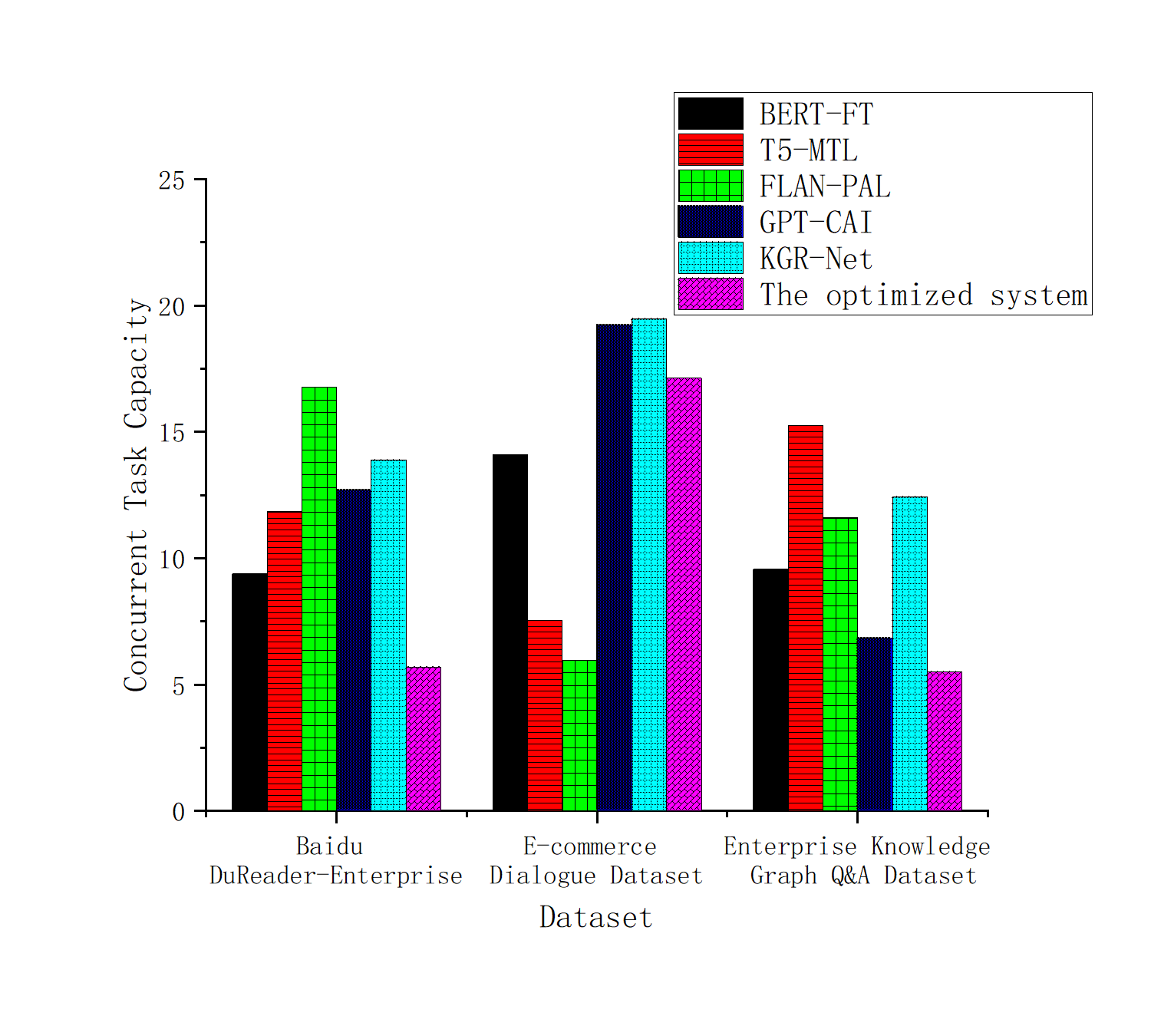

Supplement: S3 Fig — (ZIP) [file pone.0340964.s003.zip › Fig/Fig1(b).tif]

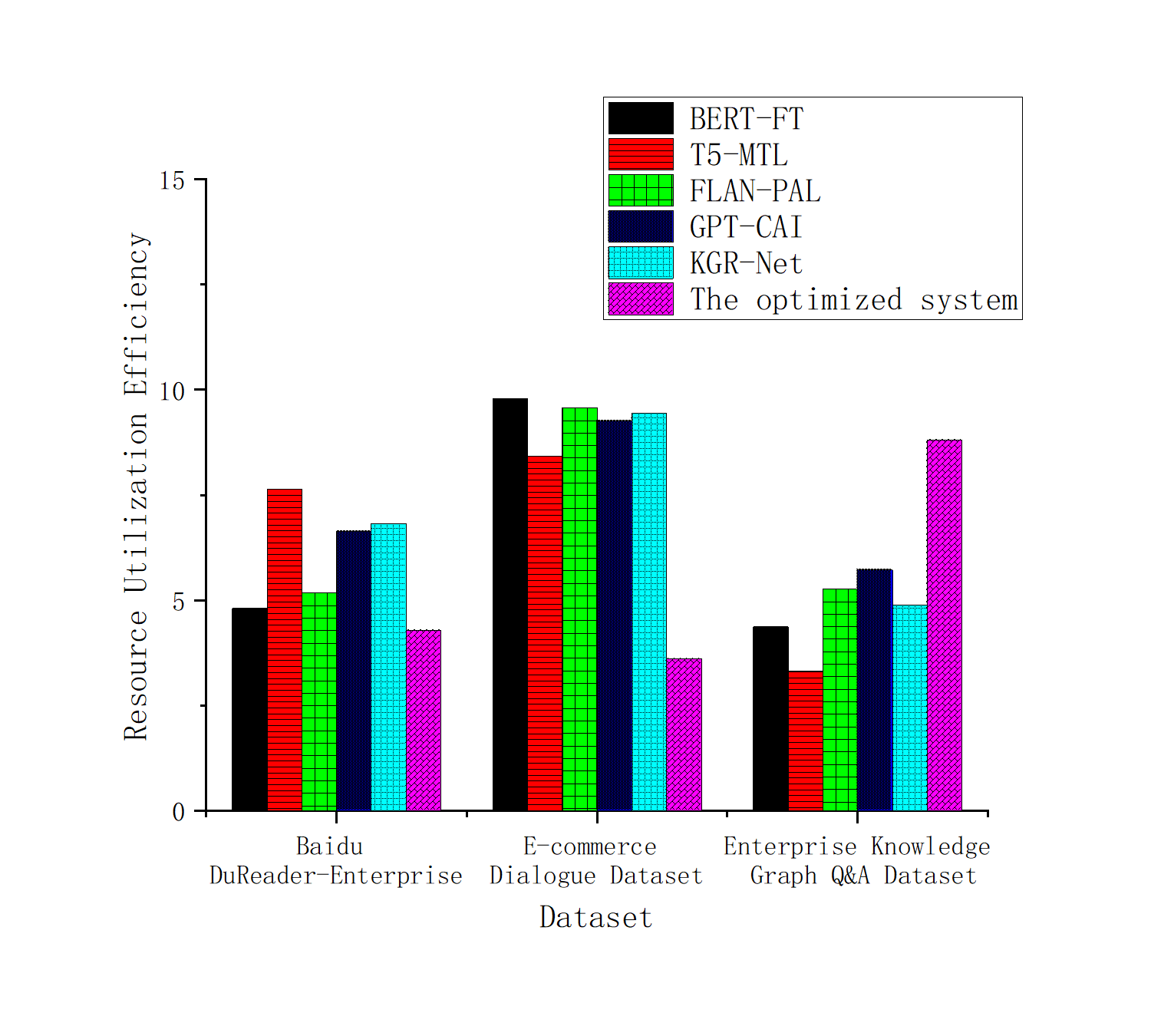

Supplement: S3 Fig — (ZIP) [file pone.0340964.s003.zip › Fig/Fig1(c).tif]

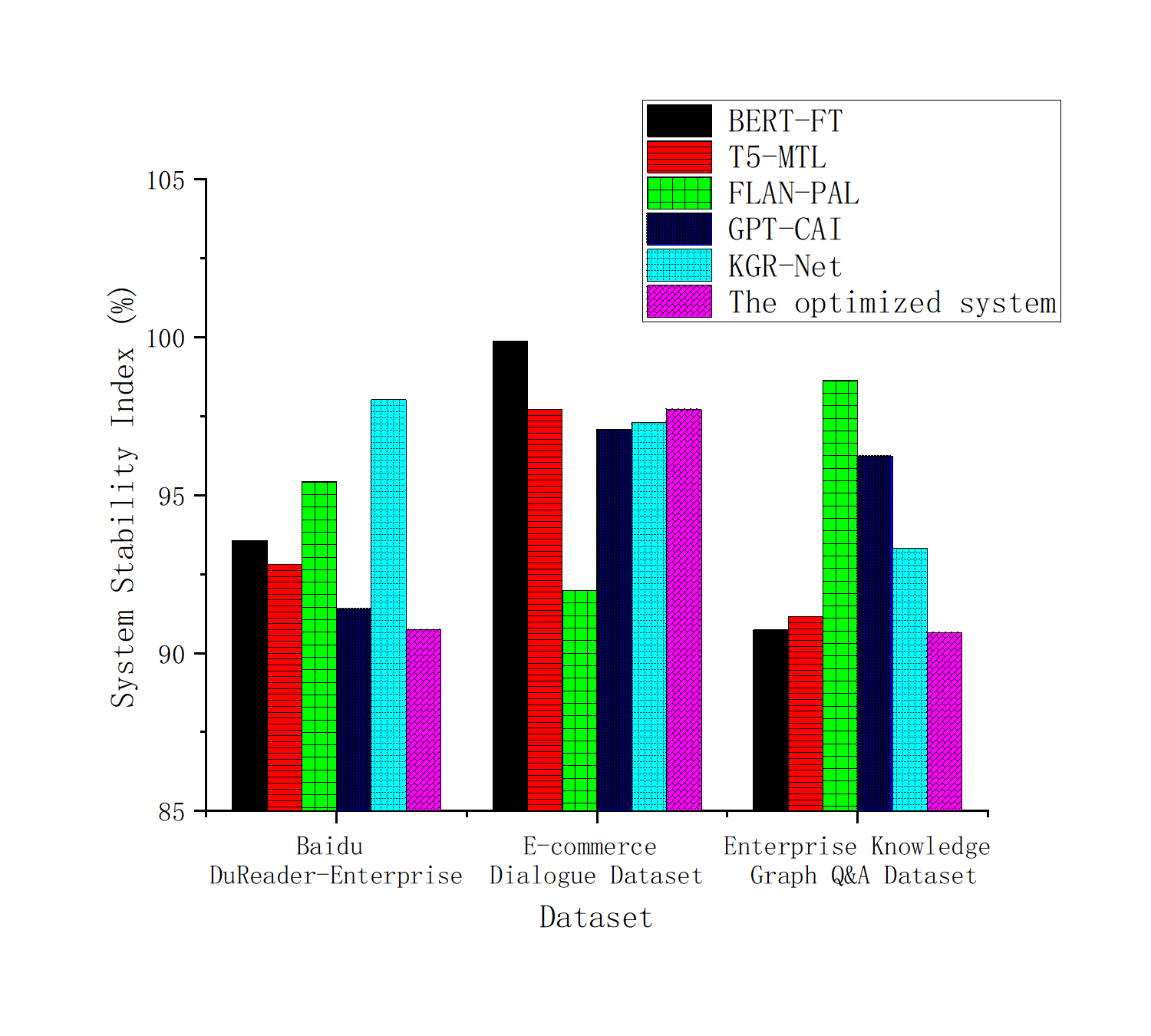

Supplement: S3 Fig — (ZIP) [file pone.0340964.s003.zip › Fig/Fig1(d).tif]

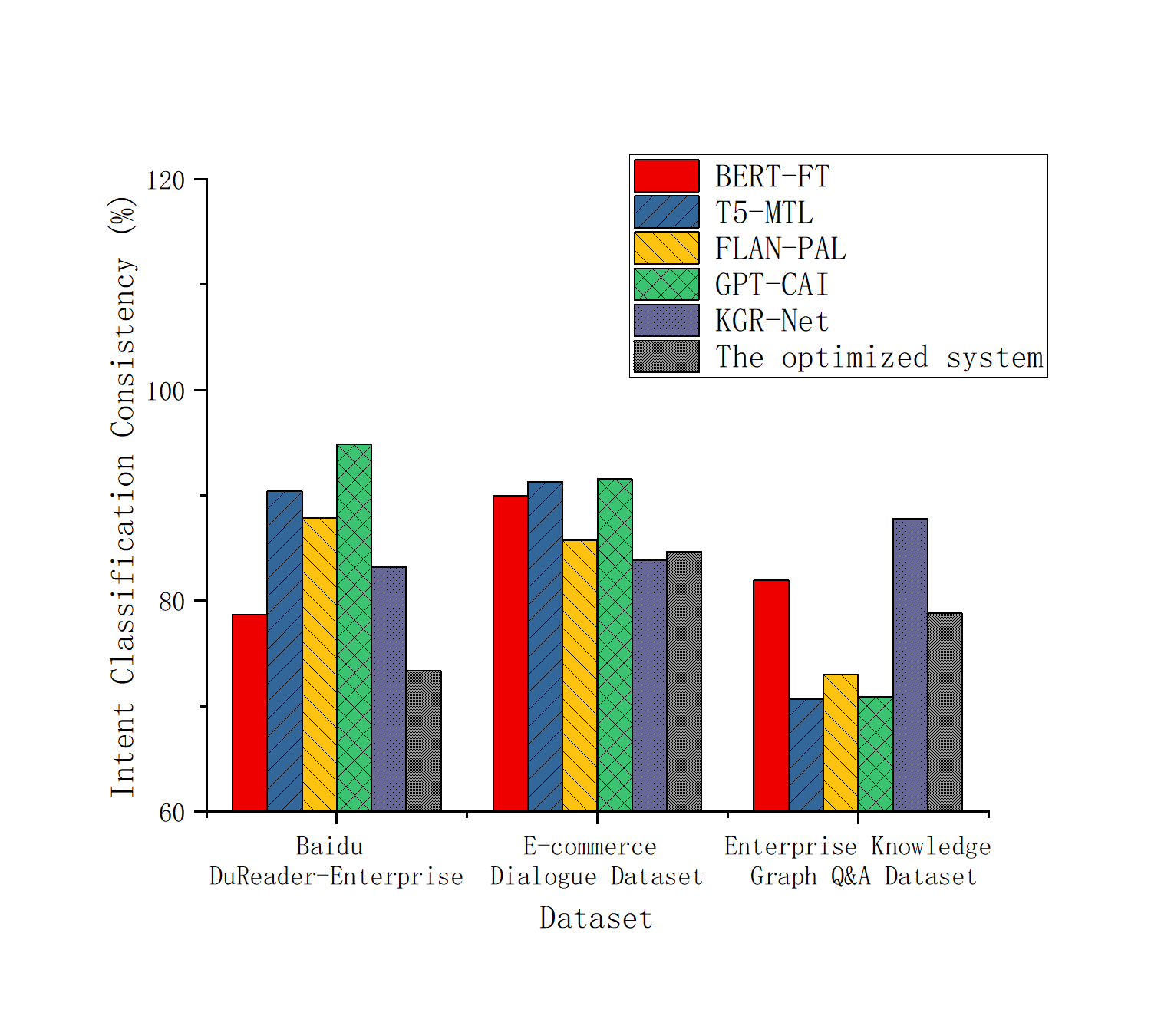

Supplement: S3 Fig — (ZIP) [file pone.0340964.s003.zip › Fig/Fig2(a).tif]

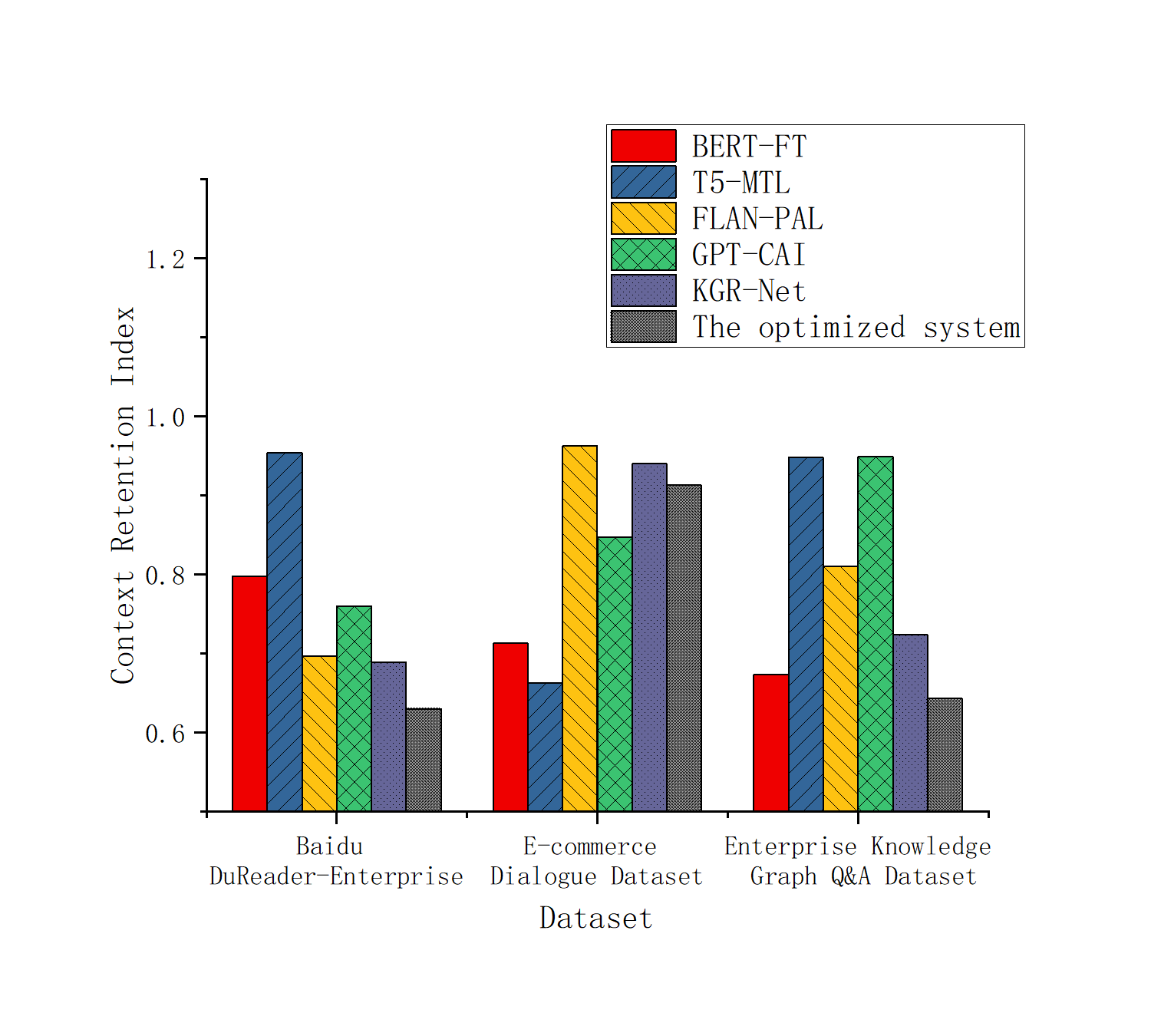

Supplement: S3 Fig — (ZIP) [file pone.0340964.s003.zip › Fig/Fig2(b).tif]

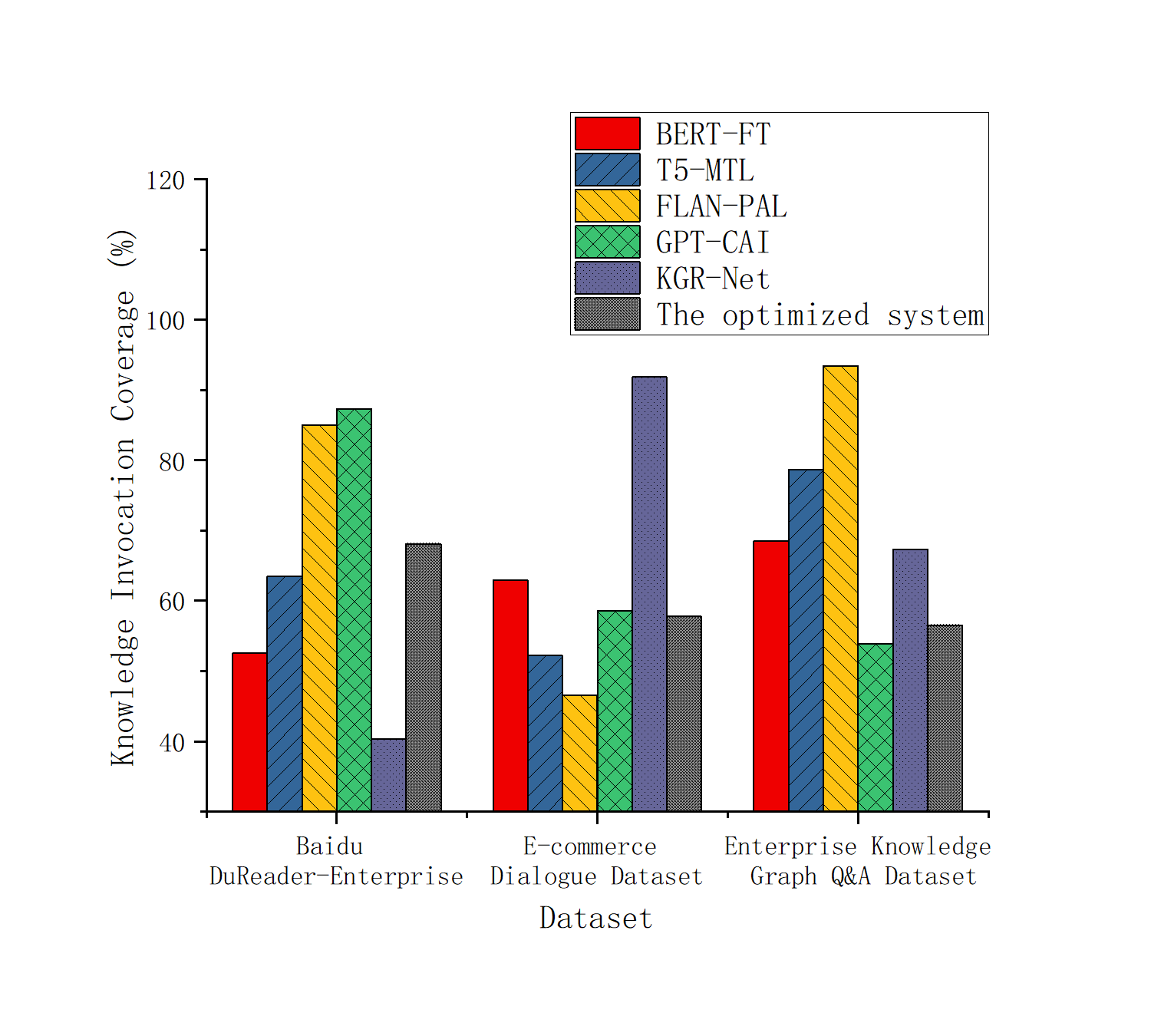

Supplement: S3 Fig — (ZIP) [file pone.0340964.s003.zip › Fig/Fig2(c).tif]

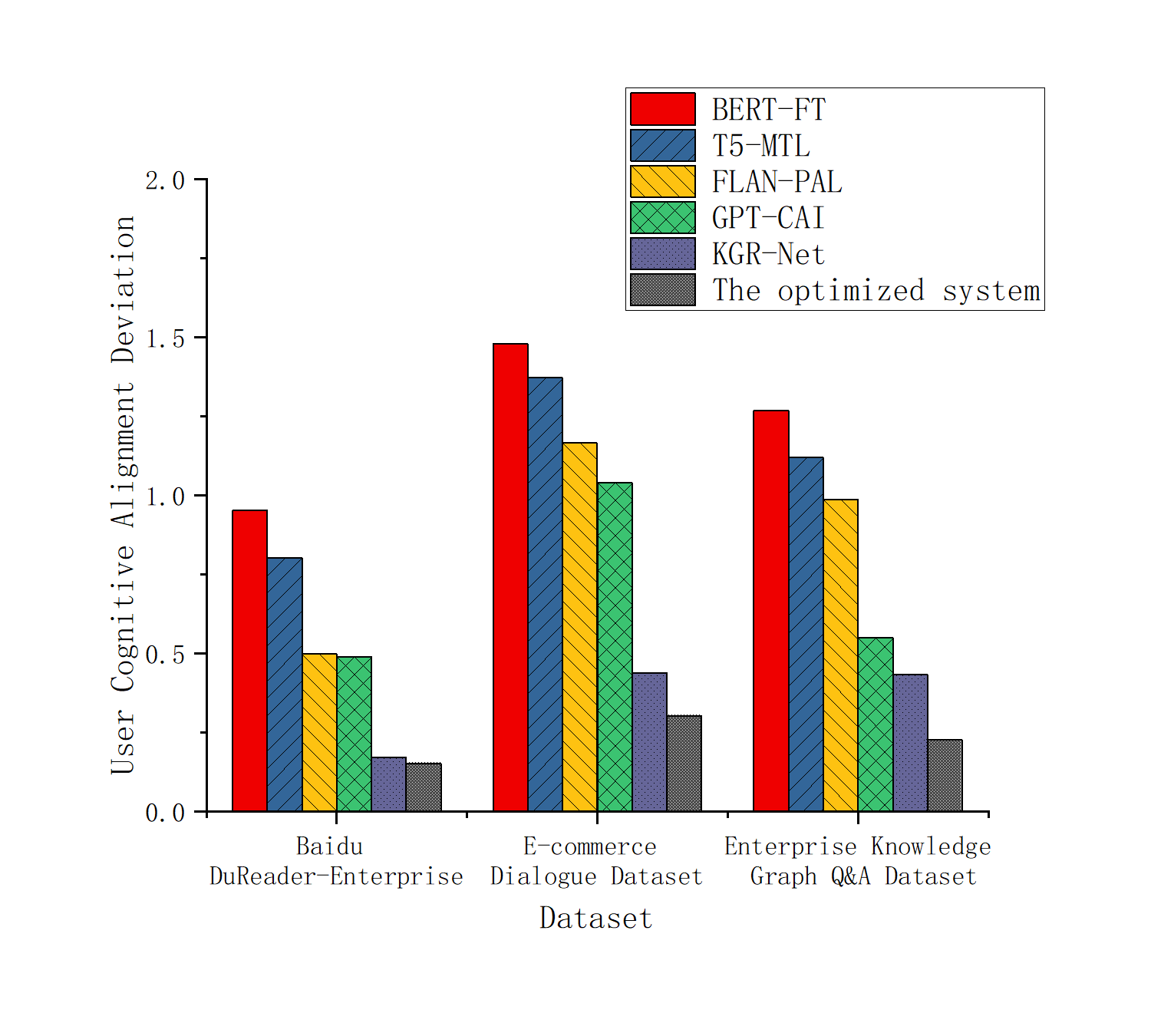

Supplement: S3 Fig — (ZIP) [file pone.0340964.s003.zip › Fig/Fig2(d).tif]
